# Household Response to Inadequate Sewerage and Garbage Collection Services in Abuja, Nigeria

**DOI:** 10.1155/2017/5314840

**Published:** 2017-05-29

**Authors:** Ismaila Rimi Abubakar

**Affiliations:** College of Architecture and Planning, University of Dammam, P.O. Box 2397, Dammam 31451, Saudi Arabia

## Abstract

Provision of sanitation and garbage collection services is an important and yet challenging issue in the rapidly growing cities of developing countries, with significant human health and environmental sustainability implications. Although a growing number of studies have investigated the consequences of inadequate delivery of basic urban services in developing countries, few studies have examined how households cope with the problems. Using the Exit, Voice, Loyalty, and Neglect (EVLN) model, this article explores how households respond to inadequate sewerage and garbage collection services in Abuja, Nigeria. Based on a qualitative study, data were gathered from in-depth interviews with sixty households, complemented with personal observation. The findings from grounded analysis indicated that majority (62%) and about half (55%) of the respondents have utilized the informal sector for sewerage services and garbage collection, respectively, to supplement the services provided by the city. While 68% of the respondents reported investing their personal resources to improve the delivery of existing sewerage services, half (53%) have collectively complained to the utility agency and few (22%) have neglected the problems. The paper concludes by discussing the public health and environmental sustainability implications of the findings.

## 1. Introduction

Provision of adequate sanitation and garbage collection services to city residents is an increasingly important public health, livelihood, and policy issue. The 2030 Agenda for Sustainable Development reaffirms UN's commitment concerning the human right to improved sanitation facilities, which are facilities that hygienically separate excreta from human contact and are used by only members of one household: toilet that flushes to sewage system or septic tank, VIP latrine, pit latrine with slab, and composting toilet [1]. On the other hand, garbage collection which involves evacuation, sorting, and safe disposal of solid waste is one of the vital public services and a useful proxy indicator of good governance [2]. The delivery of urban services can be defined as the act of ensuring that services are available to end users, including decisions about quantity and quality of services to be delivered [3]. Nonetheless, numerous cities in developing countries are unable to adequately and efficiently provide basic sanitation and garbage collection services [2–9]. In 2015, only 38% of the population in the least developed countries had access to improved sanitation facilities [1]. Even in cities where such facilities are available, they are poorly operated and maintained, thus exposing residents to serious human and environmental health risks [1, 6, 7]. Similarly, several cities in developing countries face a “garbage crisis” where between a third and one-half of generated garbage is not collected, which ends up in open spaces, in the streets and inside gutters, thus contributing to environmental pollution, flooding, breeding of rodent and insect vectors, and spreading of diseases [2, 5, 8].

As such, households respond to the problems of inadequate delivery of sanitation and garbage collection services using several coping strategies. These strategies include complaining to utility agencies to improve service delivery, relocating to another area with better level of services, changing service provider, obtaining services from alternative sources such as garbage disposal by informal collectors or household members, patiently waiting and hoping for the situation to improve, and doing nothing about the situation because of apathy or lack of options [2, 4–11]. These response strategies can be conceptualized using the Exit, Voice, Loyalty, and Neglect (EVLN) model [12], as one of the most popular frameworks of explaining households' behavioral responses to dissatisfaction with urban services delivery. The model is an extension of the Exit, Voice, and Loyalty (EVL) framework developed by Hirschman [13] who postulated that consumers, employees, or citizens respond to decline of the quality of products and services through at least one of the following three responses.* Exit* refers to terminating their relationship with the firm, organization, or state.* Voice* connotes an attempt to improve the situation through complaints and activism.* Loyalty* is the feeling of attachment to the system, product, or service, which influences the decision to either exit or voice. It improves the relationship by discouraging exit while encouraging voice.

Rusbult et al. [14] extended the EVL framework by adding* “neglect”* as the fourth response strategy, which refers to doing nothing to improve the situation due to apathy or cynicism. The EVLN model [11] was later developed to explain responses to dissatisfaction with public policies, including the delivery of public services. The framework also categorized the four responses along two dimensions: active-passive and constructive-destructive. While exit and voice are considered as “active” responses as action is involved, loyalty and neglect are regarded as “passive” responses because dissatisfied individuals just do nothing to change the situation. Similarly, voice and loyalty are regarded as “constructive” responses for they are meant to sustain or revitalize the situation. Exit and neglect, on the other hand, are considered “destructive” for they are not intended to halt the deteriorating situation but to flee from it.

The present study applies the EVLN framework to explore household response strategies to inadequate sewerage and garbage collection services in Abuja, Nigeria. The justification for using the framework is that (a) it is one of the popular models for explaining user response to dissatisfaction with services provision, which numerous studies have utilized for the past three decades [15–17] and (b) it has been applied largely in the market economies of the West and to the best of our knowledge no study has investigated its applicability in the rapidly growing cities of developing countries. Thus, this study explores how well the framework fits within the context of informality, public monopoly in basic services delivery, and bureaucratic inefficiency. The study also investigates the relative efficacy of the strategies towards enhancing the delivery of existing services or in providing substitute services. The efficacy of a coping strategy substantially matters in future household decision on which strategy to choose. Sewerage services here refer to collection of wastewater from homes and transporting it to a facility where it is treated and its eventual disposal, as well as sewer maintenance and repairs. On the other hand, garbage collection services refer to provision of bin/bag for garbage storage, collection of garbage at stipulated times, and its transportation and safe disposal.

Likewise, since most EVLN studies are based on survey methods, it is imperative to employ qualitative research to gain in-depth understanding of household responses from their own perspectives. Another contribution of the study is to underscore that while coping strategies do provide households with alternatives when public services are inadequate or absent, there are many negative consequences associated with their utilization. Several strategies are expensive and inconvenient, some cause households economic and productivity loss while searching for or obtaining alternative services, and a few have severe public and environmental health risks [2, 7–11]. In addition, most studies on urban services delivery tend to concentrate on accessibility and performance, rather than on how households respond to inadequate service delivery [14].

Abuja was chosen as a study area because it is one of the fastest growing cities in Africa. Moreover, the city is currently facing problems of frequent sewer blockages, sanitary sewer overflow (SSO) [18–20], and uncollected garbage that litters the streets and blocks drainages [18, 21, 22]. This study was conceived because a thorough search of available literature indicated that no study was undertaken to investigate how households cope with these problems in Abuja. The remainder of the article is organized as follows. The next section describes the research methodology.  Section 3 presents and discusses the study findings, which is followed by the implications in Section 4. The paper concludes with Section 5.

## 2. Material and Methods

### 2.1. Study Area

**Figure 1:**
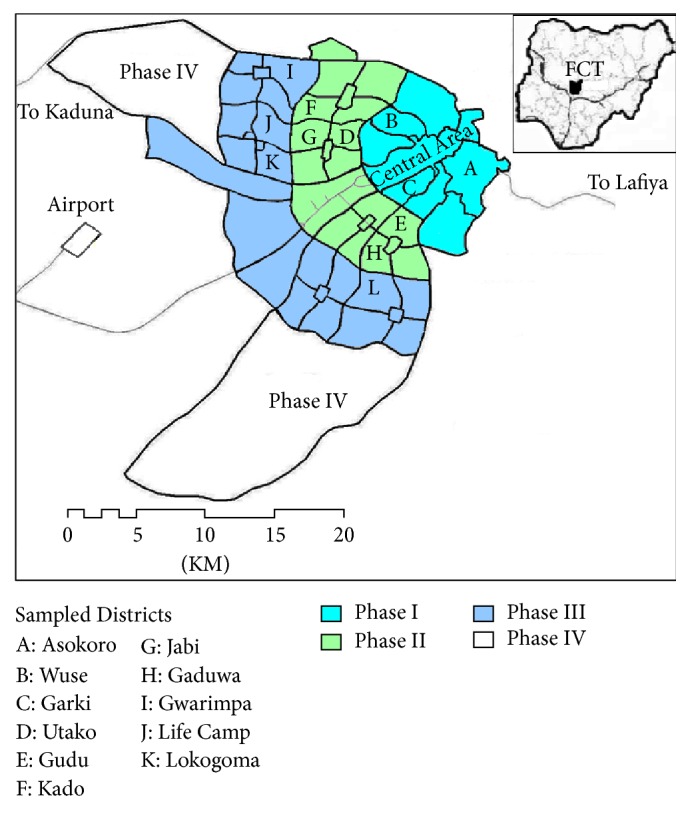
Four phases of Abuja and the sampled residential districts (source: author).

Abuja city replaced Lagos as the federal capital of Nigeria in 1991 due to latter's problems that include overcrowding, poor housing condition, inadequate basic services, and dilapidated infrastructure. Abuja is in the Federal Capital Territory (FCT), located at the geographical center of Nigeria, so that it will be readily accessible from various parts of the country. With an estimated population of over 3 million, the city is currently facing huge urbanization challenges, including the delivery of garbage collection and sewerage services due to financial and institutional constraints, as well as rapid in-migration of people due to perceived employment opportunities [4, 18]. For effective urban management and service delivery, the Abuja Master Plan divided the city into four spatially defined phases, each containing several residential districts (Figure 1). The master plan proposed 79 residential districts for the city, out of which 24 have been developed so far [18]. Abuja Environmental Protection Board (AEPB) is the agency that provides sewerage and garbage collection services in the city and the entire FCT. The utility agency owns and directly operates and maintains the city's central sewerage system. In 2004 however, the agency contracted out garbage collection and transportation to dump sites at the outskirt of the city to private contractors through a public-private partnership (PPP) scheme. In the partnership, the agency collects service charges from households and pays the contractors. As mentioned earlier, frequent sewer blockage and leakage of untreated sewage into streets are some of the current problems with the sewerage system [18, 20]. Solid waste management problems include delay or failing to collect garbage and dumping of garbage alongside roads, inside drainage channels, and in open spaces [18, 21, 22].

### 2.2. Data Collection and Analysis

This study is based on qualitative research where data was collected from in-depth interviews and direct observation. Qualitative research, which is a mainstream form of research in urban studies, was selected because of the following reasons. First, interviews capture the richness of households' everyday experience and provide context within which an event occurs. Second, service delivery is an ongoing phenomenon that cannot be manipulated, hence more appropriately studied using interview or survey techniques. Lastly, qualitative research allows cross verification of evidence from multiple sources (interviews and observation), which is a renowned technique for improving the validity and reliability of study findings [23]. As such, between May and July, 2015, the author conducted face-to-face interviews with 60 household heads or their spouses being key consumers of public services. The sample size is more than the recommended (30–45) for a qualitative research [23].

By means of key informants and snowball sampling techniques, households were selected from 12 residential districts that local planning officials and author's experience indicated are facing serious problems with sewerage and garbage collection services (Figure 1). At least three districts were chosen from each phase to ensure geographical spread and representation of both new and old areas. Eighteen key informants, comprised of fifteen household heads and three community leaders, who have stayed in the sampled districts for at least ten years were identified with the help of local planning officials. Then through snowball technique each informant was interviewed and asked to recommend other household heads or their spouses living in the same district, who were in turn interviewed and asked to recommend other participants until no additional information came out of the interviews.

The interview questionnaire, consisting of open-ended questions, was reviewed by colleagues, pretested in pilot interviews, and improved using reviewers' comments and outcome of the pilot interviews. The semistructured interviews lasted for an average of 35 minutes. The questions solicited households' experience with the delivery of sewerage and garbage collection services, how they usually cope with inadequate or lack of both services, and the efficacy of their coping strategies towards solving the problems. Additional follow-up questions were asked for details and clarifications. All interviewees voluntarily participated in the study and have granted their permissions for recording the interviews.

All the interviews were transcribed and analyzed using grounded theory, which is an approach of exploring concepts from the relationship between the gathered data and the evolving themes [23]. The analysis involved open coding of the transcribed data into three categories: action, condition, and consequence. Secondly, through axial coding these categories of codes were analyzed to find the connections (relationships, cause, and effects) between them. Lastly, selective coding involved developing themes that link and synthesize these categories of codes. It entailed iterating throughout the data to validate the emerging themes or discover new angles until no further information surfaced. Personal observations, recorded via photography and written notes, as well as review of secondary data were used to contextualize and corroborate the interview findings, thus providing a converging of line of inquiry [23].

### 2.3. General Characteristics of Interviewed Households

Table 1 shows that close to three-quarters (73%) of the interviewees were males. Length of residency varied from three to 28 years (mean = 9); higher value reflects the richness of households' experience, which also corresponds with the age of the districts. While 28% of the interviewees were living in single family housing, 72% were living in apartments. About 38% of the respondents were living in Phase I, 33% were living in Phase II, and the rest (28%) were staying in Phase III. Phase IV, located at the two ends of the crescent form of the city, was not planned in the master plan but reserved for future city expansion. Here, infrastructure is currently being developed via site and services scheme by the city in partnership with private developers [18]. About half of the households (53%) were renters, and 63% were public sector employees, which is in line with Abuja's function as a predominantly administrative city.

Out of the twelve selected residential districts, Garki, Asokoro, and Wuse are in Phase I, which is the oldest part of the city and contains the Central Area that consists of the CBD, the seat of the federal government, foreign embassies, international agencies, and several business corporations, as well as five residential districts for Abuja's elites. In Phase II, the districts of Utako, Jabi, Kado, Gaduwa, and Gudu were selected, which are high density areas for low- to medium-income people. The first three districts have been fully developed while the development of the remaining two is ongoing. In Phase III, Gwarimpa, Life Camp, Nbora, and Lokogoma districts were chosen; the first two are medium density areas inhabited mostly by senior civil servants of the FCT, while Nbora and Lokogoma districts consist of mostly residential estates developed by real estate companies for middle and upper class workers of both private and public sectors.

## 3. Results and Discussion

This section discusses how households cope with inadequate sewerage and garbage collection services and their perceptions of the efficacy of the response strategies, using the EVLN framework.

### 3.1. Exit Strategies for Responding to Inadequate Sewerage and Garbage Collection Services

Exit strategies are responses to inadequate service delivery by relocating to a jurisdiction with more satisfactory services (geographical or Tiebout exit), as well as exiting or intending to do so to a rival service provider (provider exit) [16]. Two other forms of exit have also been identified in a public monopoly where complete exit is not an option. First, the “quasi-exit” connotes temporarily obtaining essential public goods and services from the informal sector [24]. Second, “entrepreneurial exit” involves household members providing their own alternative services [25]. In this study households have utilized all these kinds of exit strategies in response to inadequate sewerage and garbage collection services, with strategies from the informal sector being dominant (Table 2).

In terms of geographical exit, only one tenant has relocated from one district to another because of persisting sewer blockage and SSO and another was contemplating to do so. Respondents generally indicated that the priority placed on getting affordable housing in Abuja makes residential relocation due to poor services less likely, as the following quote illustrates:

You know why this thing [moving] will not happen in Abuja here is because houses are generally scarce. Whether you like it or not, you have to stay. Because where are the houses in the first place? People are even living in the outskirt of the town and are suffering there. So, there is no way you can leave your house because of sewage or refuse. If the rent is right everyone will stay. You have to continue to bear with them until they provided. In fact, you have no option of moving to anywhere, that is just it.

Geographical exit is the least utilized form of exit (3%) in this study, which is not unexpected given that even in the developed countries people do not usually change residence due to inadequate service delivery [26]. For example, in a survey of user response to public services delivery in Chicago, garbage collection problems were not part of the main determinants of participants' intention to change residence [15]. Similarly, dissatisfaction with public services has weak relationship with geographical exit in the UK [16]. This is despite more opportunities for geographical exit in the developed countries, which include more accessible mortgage financing and employment opportunities in both private and public sectors that are distributed throughout states and counties. Conversely, employment and residential mobility in developing countries are restrained by urban bias, deep-seated sociocultural bonds to localities, and strict residency regulations throughout different states and localities. In brief, geographical exit is possible only if its benefits exceed its costs: money, time, and energy required for relocation [27].

Concerning provider exit that involves switching from one service provider to another, it is feasible only in a pluralistic supply of services. In Abuja, although the sewer infrastructure is owned by AEPB, repairs can be done through the utility agency or the private sector depending on the extent and location of the problem. In this study, 37 households (62%) have hired commercial plumbers for sewer repairs instead of going to the utility agency. They indicated that repairs in the informal sector are more effective in addressing the problems because they are faster, last longer, and are even cheaper than repairs done by the agency:

When you get a private [person] and you pay him, he will do it up to your satisfaction. Unlike the government [staff], which you do not have control over them. They will say they are coming and you would not see them for another month and there is nothing you can do about it. You continue to follow them, begging them to come and do their job.

**Figure 2:**
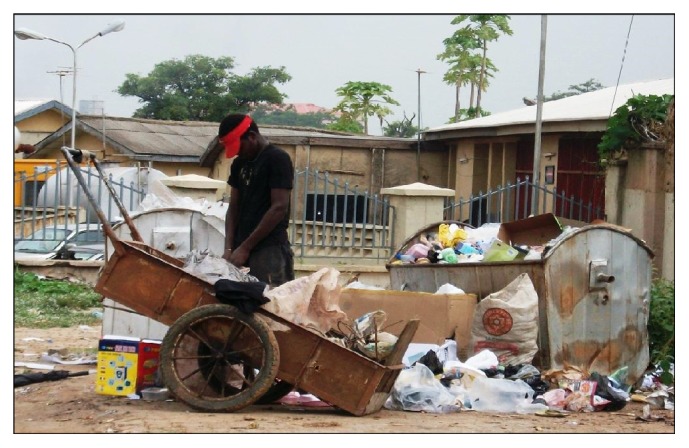
Scavenger collecting garbage in Utako district, Abuja (Source: field work, 2015).

Because garbage is collected by contractors through the partnership, there were six households (10%) that switched from a “disappointing” to a “better” garbage contractor. The households maintained that this kind of exit strategy was effective in solving garbage collection problems. On the other hand, 25% of households have employed entrepreneurial exit where they used their cars or sent their children to dispose garbage, usually in bigger bins at nearby commercial centers or indiscriminately in any convenient place. With regard to quasi-exit, eight households (13%) have hired laborers from the informal sector to dispose sewage from their septic tanks, instead of through the utility agency. Likewise, 33 households (55%) have cited hiring scavengers for garbage disposal whenever it has not been picked. Thus, despite contracting out garbage collection to private contractors, interviewees and personal observation indicated that scavengers still collect garbage in several parts of Abuja (Figure 2).

Some respondents maintained that garbage collection by scavengers is cheap and immediate as the scavengers can be found nearby. The scavengers while providing alternative services also depend on collecting recyclable materials for their livelihood [28]. Indeed, if the scavengers are employed by the contractors, the response and efficiency of garbage collection could be improved. In addition, they will have formal jobs thereby escaping constant harassment from the city, as well as improved working condition, such as wearing of kits like boots and hand globes. This implies that an issue worth further investigating is whether AEPB is effectively monitoring the performance of the companies involved in the partnership and whether there is a sanction that could be applied to any contractor that is not delivering required services.

Exit is considered as a destructive response because the affected utility agency could face customer and revenue decline, not knowing the problems causing exit which could be useful in improving services, as well as less competition or eventual monopoly that could result from decline or demise of services [15]. In general, exit strategies depend on users' ability to evaluate service quality, multiple service delivery, the costs of carrying out exit, and lack of investments like homes, social capital such as family and friends, or sentiment that might dissuade users from exiting [27]. In market economies where private service providers compete for customers, exit can help improve service performance given that business profit depends on the level of customer patronage.

### 3.2. Voice Strategies for Responding to Inadequate Sewerage and Garbage Collection Services

**Figure 3:**
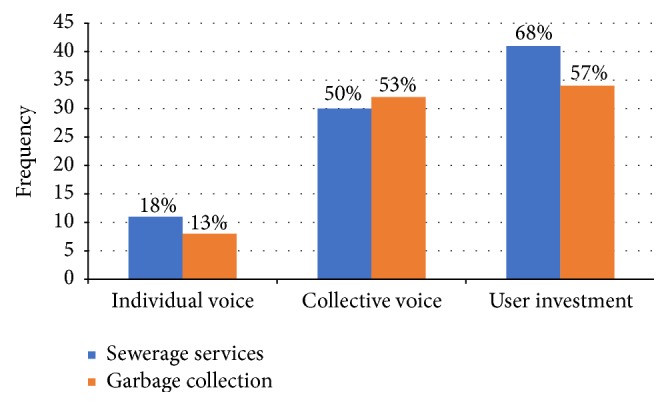
Households' voice responses to inadequate service delivery.

Any response to inadequate services is generally considered to be a voice if it is intended to improve service delivery and not to flee from problems. It includes complaints, demonstrations, campaigning, or community pressure aimed at getting improved services [12]. Dowding and John [16] classified voice into “individual” and “collective” voice and later Dowding et al. [27] introduced “investment” (investing resources to improve service delivery) as another form of voice. While in the present study there is no evidence of voice through demonstration and campaigning, households have individually and collectively complained to AEPB and have also invested their own resources to improve the delivery of the existing sewerage and garbage collection services (Figure 3).

Individual voice refers to complaint by households singularly. In this study, only eleven (18%) and eight (13%) households have complained individually to AEPB about inadequate sewerage and garbage collection services, respectively. They indicated that individual complaints are often not effective in solving problems because of bureaucratic delays, lack of response, and the need to part with extra money. Some interviewees mentioned several incidents where AEPB responded to complaints about sewer blockage or SSO only after several weeks. In general, individual households, except wealthy or those with strong political power, usually have less power compared with a group of people to ensure bureaucratic responsiveness.

On the other hand, the collective voice represents actions by groups of neighbors or organizations when they act jointly to improve service performance. This strategy has been utilized by 30 (50%) and 32 (53%) households when they complained about sewerage and garbage collection problems, respectively. The mode of complaint was through their community based organizations (CBOs), or by delegating some residents to formally complain to the agency. They indicated that using CBOs as a medium of complaint is effective in solving problems as the organizations have the required resources, such as money and elite membership to induce the authorities to tackle the problems, which could even involve taking legal action. The likely reason for more utilization of collective than individual complain is that the first is louder than the latter; thus the utility agency is prone to respond more promptly to collective voice.

In terms of the modes of complaints, respondents indicated that complaining in person is more effective in addressing a problem than phone calls or writing mails, given that acquaintance with the customer service personnel improves the chances of the complaints being resolved. Many telephone calls to the agency were less effective because of frequent breakdown of communication. Emailing the agency was also less effective as emails are usually replied by an automated message that customer complaint has been received and a staff will consider it and that is often the end of the matter. The mass media is another kind of collective voice that is effective in making the utility agency attend to customer complaints. Some local newspapers and radio and TV stations publish news articles and broadcast interviews about inadequate service delivery in the city and households' coping mechanism. As found in the present study, the use of the media as a form of voice was evident in Accra, Ghana, where FM radio stations are playing significant role in improving service delivery by ensuring that the voices of the poor and marginalized are heard [29]. An interesting pattern in this result is that, except in the case of complaints by groups of neighbors, there had been more complaints about sewerage than garbage collection. This suggests that either there were more problems with the sewerage services than garbage collection, or respondents were more concerned with the former than the latter. Similar to this study, a survey of Chicago residents found that of those that reported high level of dissatisfaction with garbage collection only one out of five had complained about the problem [15].

Another kind of voice is user investments to improve service delivery. In this study 41 (68%) and 34 (57%) households reported investing their money and time to improve the delivery of sewerage and garbage collection services, respectively. They indicated that although the infrastructure is owned by the state and they pay their bills when due, they had in many incidences collectively contributed money to repair broken sewer pipes, or even gone to the extent of replacing smaller with higher capacity pipes:* “before, we were using 2 cm pipe, so we upgraded it to bigger pipe so that even if something goes in, it will not block.” *Thus, instead of complaining to the utility agency and wait in perpetuity for repairs and maintenance works, households indicated that they often performed the duty of the government by collaborating to execute projects for maintaining and improving service delivery. However, there is a concern about the quality of the work done informally, as well as increasing load on the existing infrastructure. Likewise, some households have donated money to fuel garbage trucks that serve their communities, while others have purchased garbage bins and bags from the market because they have not been provided. These kinds of coping strategies are different from the classical voice in the EVLN framework because households in this situation actively contribute their resources to enhance the delivery of essential public services. These kinds of investments can be used as a measure of loyalty.

Voice responses are generally considered as constructive because they provide feedback for improving service performance, a kind of warning about a risk of exit if problems are not tackled. They are generally believed to be more effective in a competitive mode of service delivery and their utilization is influenced by socioeconomic factors [26, 30]. For example, high-income and more educated households are usually more vocal or “alert” than the poor and less educated who are ordinarily less vocal or “inert” [31]. Curtice and Stratos [32] observed that the voice by alert households benefits the inert households too, but if the first exited instead of staying to voice from within, then the latter would be “locked” to poor services as they lack the means to exit. Households who are locked in are more likely to voice than those who are not. Thus, reducing the opportunity for people to exit improves the efficacy of voice, and vice versa.

### 3.3. Loyalty Strategies for Responding to Inadequate Sewerage and Garbage Collection Services

**Figure 4:**
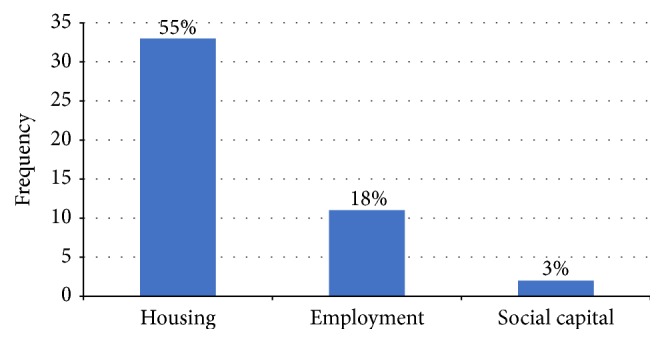
Types of household loyalty.

In the original EVLN model, loyalty refers to passively remaining with unsatisfactory or inadequate services without exiting due to optimism that the situation would improve [12]. Later, loyalty was considered as a distinctive response where households bear with inadequate services due to their investment in the area, including tangible assets like homeownership, employment, or social investments such as friends, family, and community sentiments [33]. In this study, homeownership and affordable rents were the reason given by 33 households (55%) to bear with inadequate delivering of both services in their locality, compared with 11 households (17%) who were compelled to tolerate the situation because of their jobs (Figure 4).

Although Abuja city experiences influx of migrants and job-seekers who might cherish staying close to friends and relatives, coupled with the fact that the city consists of residents from different tribes and faiths, this study found little evidence of loyalty due to social capital. Only two households (3%), both living in Utako district, indicated that though rent is cheaper in other areas, closeness to their tribesmen is the main reason for their continued stay in the district despite incessant sewerage and garbage collection problems. Probable reason for this finding is the advancement in telecommunication which minimizes the need for face-to-face interaction with relatives/friends and ethnic groups. However, some previous studies found cases of loyalty due to social capital. For example, in a survey of citizens of Scotland and Wales, Campbell et al. [31] found that higher social capital, measured as the ability to name neighbors, makes geographical exit less likely despite dissatisfaction with healthcare and education services. When faced with problems of service delivery, households with more investments are more probable to employ voice or loyalty than geographical exit or neglect.

### 3.4. Neglect Strategies for Responding to Inadequate Sewerage and Garbage Collection Services

**Figure 5:**
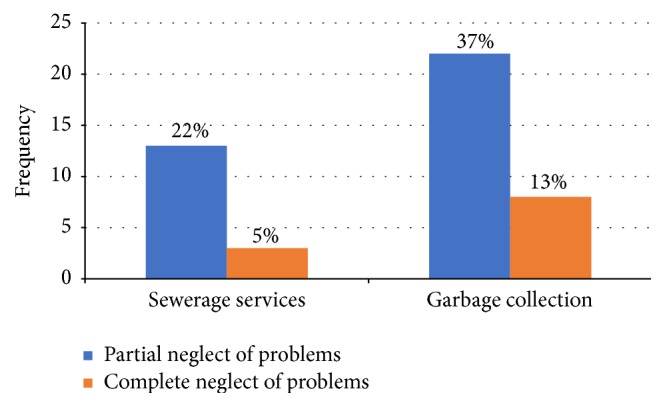
Households' neglect responses to inadequate service delivery.

Neglect connotes ignoring a problem by way of inactivity towards improving the situation or solving the problems and it includes cynicism, apathy, nonvoting, believing that the state has no effect on people's existence, and distrusting the municipal officials [11]. This strategy really depends on the urgency of the problem. Given that completely neglecting problems such as sewer blockage or SSO is very unlikely due to stench and health threats, this study modified the EVLN model by categorizing neglect into* “partial”* and* “complete”* (Figure 5). While partial neglect is defined here as lack of complaints only, complete neglect refers to the “do-nothing” behavior as postulated in the EVLN model. The partial neglect can be measured if households ignore the formal channels of addressing a problem via the utility agency or contractors, while the complete neglect is measured if both the formal and informal channels are ignored.

Thirteen (22%) and 22 (37%) households have utilized partial neglect in dealing with problems of sewerage services and garbage collection, respectively, but have employed alternative means of solving the problems. Respondents mentioned the necessity of having a functioning sanitation system, problem of stench, and risk to their health as their main reasons for seeking alternative solutions. On the other hand, 38 households (63%) indicated that they completely ignored uncollected garbage, while only three households (5%) completely neglected blocked sewers and SSO located away from their homes:* “*y*ou know when a problem is everybody's problem, it is nobody's problem. You know our culture, nobody took the responsibility to go and report about the refuse, because something is public. It is only when it affects you directly.” *

A noteworthy observation from Figure 5 is that the frequency of neglect for sewerage problems was less than that for garbage collection, likely because the consequences of neglecting broken sewer system are higher than that of uncollected garbage. Similarly, this study uncovers other kinds of neglect responses that include nonpayment of utility bills and refusing to report problems to the utility agency, which are not intended to improve the situation but could lead to its deterioration. According to author's observation and experience, these kinds of neglects are common responses to inadequate delivery of urban services in the city. According to Jilke ([30], p.5) one of the key aspects of providing essential public services is that “the classical exit option of completely withdrawing from the service in question is often not feasible, too difficult, or associated with extremely high costs.”

## 4. Implications of the Study Findings

### 4.1. Environmental and Public Health Implications

This study espouses some key environmental and public health implications of its findings. First, household utilization of some coping strategies could lead to water pollution and land degradation. Although lakes and rivers provide avenues for pleasure and for recreational aspects of urban culture, a respondent living in Kado district mentioned that some trucks that evacuate sewage from septic tanks do empty it into Jabi Lake or nearby streams. Likewise, one respondent has observed a neighbor evacuating sewage from septic tank and discharging it into storm water drainage when the rain was falling. Similarly, garbage collected by scavengers is often disposed at any convenient place like water bodies, empty plots, or construction sites. When garbage has not been collected by the contractors, many respondents have admitted that they or their household members have haphazardly disposed it inside storm drainage and on roadsides, building construction sites, parks, and vacant lots, as testified by one respondent:

I just put the refuse in a black bag and drive to another place to dump or throw it into the bush. That is where people always throw their refuse, so you don't even notice refuse. Some also dump it in the lake or river.

These coping practices are detrimental to the environment, aquatic animals, and public health. For instance, the same Jabi Lake that respondents in this study reported being polluted with garbage and untreated sewage is the one that another study observed water vendors fetching water from [34]. This could expose the people to acute communicable diseases such as diarrhea, cholera, dysentery, typhoid, and hepatitis A. The cost of water contamination from improper waste disposal has been put at ₦10 billion (~US $33 million) annually and putting the lives of about 40 million Nigerians at risk [2]. Other studies have also associated improper waste management practices with health risks, especially among women and children, who are burdened with carrying garbage often on their heads, being late at school, and possible physical harm when disposing garbage away from homes [4, 8, 35].

Second, employing these coping strategies can result to air pollution and foul smell. When garbage has not been picked by the contractors, households often gather the combustible materials and burn them in an open fire, which has caused one incident of fire outbreak during the dry, windy season. Although open burning of garbage done at the household level is not on a large scale, its negative environmental and human health impacts include foul odor, atmospheric pollution, release of greenhouse gasses, and smoke that lowers the aesthetic quality of the landscape [8, 9].

Third, some of these coping strategies pose severe public health risks. Indiscriminate dumping of garbage leads to a dirty environment that harbors flies and rodents, in addition to contamination of surface and underground water. Through household coping mechanism, sewage is often directly emptied into the surface water or into storm drainage where it mixes with storm water and flows into water bodies. These pose threats to public health and place increasing demand on the already overwhelmed healthcare system of the city. Instead of this attitude, the wastewater, if adequately treated, can provide reliable water and nutrients for maintaining the city's open spaces and parks that constituted 32% of its land use [18]. Whereas poor sanitation practices cause about 280,000 diarrheal deaths annually in the world, in Africa about 115 people die every hour from diseases related to poor sanitation, poor hygiene, and using unsafe water [36]. In Nigeria, children under 5 years old have 38% higher risk of dying from poor sanitation and unsafe water sources [37]. In view of these implications, citizens need to be enlightened that there are severe health risks associated with every stage of liquid and solid waste handling, treatment, and disposal. Improper handling of waste could expose them to hazardous substances and can contaminate water, soil, and food on the farmland, while open burning of garbage contributes to greenhouse gas emissions [7]. Certainly, facilitating access to improved sanitation facilities has great health benefits, including reduction in diarrhea risk by up to 37% and schistosomiasis by up to 77% [6].

### 4.2. Implications on the EVLN Framework

There are five major implications of the findings on the EVLN framework. First, quasi and entrepreneurial exits and user investment were found as the dominant responses to inadequate sewerage and garbage collection services. Households have relied mainly on the informal sector to obtain alternate services (quasi-exit) and invested their resources to enhance the delivery of basic services. This became necessary when complaints were not successfully addressed by the utility agency and switching to other services and relocating to another part of the city are not options due to monopoly and dearth of housing options. On the other hand, neglecting basic services is impossible due to necessity. Thus, the PPP in garbage collection in Abuja appears wanting in service delivery. Inadequate monitoring and selecting unqualified contractors are among the problems undermining the partnership [18, 21, 38]. As such, the agency should do benchmarking and set performance indicators they can regularly monitor. Thus, this resonates on the broader theoretical debates on the efficacy of privatization mode of public service provision in Nigeria and other developing countries. These self-help efforts also suggest residents' demand for more improved services. Other modes of service delivery such as community-driven development that were reported to be effective in several developing countries should be considered. For example, the CBOs can form cooperatives that will be involved in garbage collection similar to the contractors [10]. Alternatively, community representatives can be involved in decision making related to service delivery, including recruiting of contractors and in performance monitoring [9]. Engaging the local community could also encourage more constructive responses like proper handling of garbage and sewer infrastructure, prompt reporting of problems, and having a feeling of ownership of the services [2, 4]. This is important because, due to urban management challenges such as high urbanization rate, governments of many developing countries are financially, institutionally, and technically incapable of delivering adequate services single-handedly.

Second, CBOs and the media were very effective means of complaints in this study. This has been underreported in previous EVLN studies. In this study, respondents reported that CBOs and mass media played a key role as a medium of voice in getting their complaints addressed by the utility agency. CBOs and the media are a very effective form of voice, especially in a democracy, for they provide the opposition politicians with a tool to challenge the ruling government to improve service delivery. While the CBOs have the resources, including elite membership, required to compel the utility agency to tackle the problems, television and radio broadcasts as well as online social media make complaints louder, draw public sympathy to the complainants, and compel utility agencies to be responsive. The latter can be likened to voice directed to “third parties” instead of the service provider [33].

Third, the author of the present study suggests splitting the neglect response into “partial” and “complete” neglect. This is because urban services are not of equal importance: sewerage and garbage collection services (and drinking water as well) are necessities of life that cannot be completely neglected—the “do-nothing” type of neglect in the EVLN model. Households could opt to not complain to utility agencies, but they must certainly do something to address such problems. The c*omplete *neglect can apply to a situation where households completely ignore minor issues like accumulated garbage or SSO on the main road. On the other hand, the* partial *neglect (lack of complaints) can refer to responding to problems like faulty sewerage (blockage/broken pipes) or uncollected garbage by not complaining to the utility agency or relocating to another area, but through self-devised strategies or the informal sector because such problems cannot be completely neglected.

Third, this study deduces that the EVLN response categories and dimensions are not mutually exclusive. As mentioned earlier, the EVLN model categorized responses into four distinct types and along active-passive and constructive-destructive dimensions. This is founded on the assumption that a response cannot belong to more than one category: all exit strategies are both destructive and active and all voice strategies are active and constructive, while all loyalty strategies are both constructive and passive and all neglect strategies are both passive and destructive. This is problematic because in some instances the responses are not mutually exclusive, like the case of contributing money to fuel garbage truck, which can be both voice and loyalty response. Similarly, the quasi-exit strategy of garbage disposal by household members or scavengers could be both destructive and constructive. It is destructive because, if improperly done, it could lead to health problems and boys/girls going to school late. It is also constructive because scavenging helps the city get rid of its garbage as many city governments in developing countries cannot single-handedly collect garbage [9].

Lastly, the low-income households are not the only “locked-in” users in monopoly provision of basic urban services. Several EVLN studies reported that because the poor and less-educated households could not effectively voice or relocate to where services are better, they are “locked” in areas with inadequate or poor service delivery [32]. However, this study found that this is not the only case with regard to basic services provided via public monopoly. Due to lack of options in a monopoly, even the rich and educated households are locked in some neighborhoods facing incessant sewer blockage and SSO when complaints could not effectively solve the problems. Similarly, due to accommodation shortages throughout Abuja, some fairly educated and middle class households living in the suburban districts where service delivery is inadequate indicated that, due to acute scarcity of housing in the central city where services are better, they had no choice but to stay in their current neighborhoods.

## 5. Conclusion

This paper has improved our understanding of how households respond to inadequate sewerage and garbage collection services in Abuja. The response strategies were predominantly from the informal sector (quasi-exit) and self-devised (entrepreneurial exits), as well as investing personal resources to improve the delivery of basic public services, suggesting less efficiency in service delivery. The study found rare geographical and provider exits because of public monopoly in service delivery and few neglect responses mainly due to the necessity of both services. With regard to the efficacy of responses, complaints by CBOs and groups of neighbors and through the media were found to be more effective than individual complaints, phone calls, or sending emails in arresting the decline in service delivery. Whereas substantial loyalty to homeownership, affordable rent, and closeness to places of businesses have been reported, there was little evidence of loyalty due to family, friends, and tribe.

This article also found that the efficacy of a response strategy in improving service delivery or in providing alternative services is the major determinant of which strategy households choose. When faced with inadequate sewerage and garbage collection services in the future, households indicated their preference for self-devised coping strategies or utilizing the informal sector more than reporting to the utility agency. This is in line with the assertion that previous experience about the success or otherwise of a strategy or the likelihood of its efficacy in enhancing service performance substantially counts in deciding strategy selection. The theoretical implications of the findings of this study include the need to split neglect into “complete” and “partial” and buttressing the arguments that the EVLN responses and dimensions are not mutually exclusive and that the poor and less-educated households are not the only locked-in service users. From the public and environmental health perspective, the implications of utilizing the informal or self-devised coping strategies include possible environmental pollution and exposing the public to health risks.

Finally, household responses to inadequate delivery of sewerage and garbage collection services in this study differ significantly from those in the developed countries. In the developed countries both geographical and provider exit options are more feasible, and complaints are more effective in improving public service delivery as utility agencies respect customer rights. However, as this study found geographical exit unfeasible and complaints less effective, households' most effective coping strategies are to turn to the informal sector or to invest their own resources to enhance the delivery of essential services. Future research should specifically assess the implementation of the partnership in solid waste management in Abuja from the perspectives of the stakeholders (AEPB, contractors and residents) to uncover obstacles that are undermining it and proffer ways of overcoming them. Similarly, in-depth study is recommended to assess the impacts of Abuja's sanitation system on human health and environmental sustainability.

## Figures and Tables

**Table 1:** Characteristics of interviewed households.

District	Households	Gender		Tenure		Housing type		Mean length of residency (years)
		Female	Male	Renters	Owners	Single-family	Apartment	
Phase I	23 (38%)	7	16	12	11	4	19	13
Phase II	20 (33%)	4	16	14	6	3	17	6
Phase III	17 (28%)	5	12	6	11	10	7	8

*Total*	**60**	**16**	**44**	**32**	**28**	**17**	**43**	**9**

**Table 2:** Households' exit responses to inadequate services delivery.

Coping strategies	Frequencies
Sewerage services	
(i) Residential relocation due to sewerage problems	2 (3%)
(ii) Sewer maintenance by the informal sector	37 (62%)
(iii) Disposal of sewage from septic tank informally	8 (13%)
Garbage collection	
(i) Changing garbage collection company	6 (10%)
(ii) Garbage disposal by scavengers	33 (55%)
(iii) Garbage disposal by household members	15 (25%)
